# Primary healthcare in transition – a qualitative study of how managers perceived a system change

**DOI:** 10.1186/1472-6963-13-382

**Published:** 2013-10-03

**Authors:** Andy Maun, Kerstin Nilsson, Carina Furåker, Jörgen Thorn

**Affiliations:** 1 Department of Public Health and Community Medicine/Primary Health Care, Institute of Medicine, The Sahlgrenska Academy, University of Gothenburg, Box 414, 405 30 Göteborg, Sweden; 2 Institute of Health and Caring Science, The Sahlgrenska Academy, University of Gothenburg, Box 457, 405 30 Göteborg, Sweden

**Keywords:** Qualitative research, Leadership, Practice management, Health care reform

## Abstract

**Background:**

Primary healthcare in Sweden has undergone widespread reforms in recent years, including freedom of choice regarding provider, freedom of establishment and increased privatisation. The key aims of the reforms were to strengthen the role of the patient and improve performance in terms of access and responsiveness. The aim of this study was to explore how managers at publicly owned primary healthcare centres perceived the transition of the primary healthcare system and the impact it has had on their work.

**Methods:**

In this qualitative study, 24 managers of publicly owned primary healthcare centres in the metropolitan region of Gothenburg were recruited. Semi-structured interviews were conducted and data were analysed using content analysis inspired by Silverman.

**Results:**

The analysis revealed two core themes: *The transition is perceived as a rapid change, enforced mainly through financial incentives* and *Prioritisation conflicts arise between patient groups with different needs, demands and levels of empowerment.* The transition has produced powerful and rapid effects that were considered to be both positive and negative. While the new financial incentives were seen as a driving force and a tool for change, they also became a stress factor due to uncertainty, competition with other primary healthcare centres and negative feelings associated with staff cutbacks. The shift in power towards the patient improved access and service but also led to more patients with unreasonable demands. Managers found it difficult to prioritise correctly between patient groups with different needs, demands and levels of empowerment and they were concerned about potentially negative effects on less empowered patients, e.g. multi-morbid patients. Managers also experienced shortcomings in their change management skills.

**Conclusions:**

This qualitative study shows the complexity of the system change and describes the different effects and perceptions of the transition from a manager’s perspective. This suggests a need for improved follow-up and control in order to monitor and govern system changes and ensure development towards a more effective and sustainable primary healthcare system.

## Background

Leadership and governance are generally considered the most complex and yet the most important functions of government in relation to the health system [1]. In Sweden, there are three independent levels of government involved in the health system – national, county and municipal – each funded primarily through tax revenue [2]. In recent years, primary healthcare in Sweden has undergone the most widespread reforms in decades. The key objectives of the reforms were to strengthen the role of the patient and improve performance in terms of access and responsiveness [3]. These aims were a response to research, which showed that a strong primary healthcare system contributes to cost effectiveness [4] and that patients demand rapid access to good primary healthcare [5]. The changes involved freedom of choice regarding the provider, combined with freedom of establishment and increased privatisation of providers [6].

The primary healthcare system in western Sweden underwent a transition from a traditional budgetary system to a comprehensive, fixed capitation payment system, through which providers receive payment for the number of registered patients and their estimated ‘illness burden’ [7]. Together with freedom of establishment, this resulted in competition between primary healthcare centres (PHCCs) [8]. There is a growing trend internationally towards the use of financial incentives to reward providers for improving the quality of primary healthcare services although a recent review study showed that there is insufficient evidence to support the use of financial incentives to improve the quality of primary healthcare [9]. As there is limited consensus on how to define, model or measure stewardship of the health system [1], it is relevant to explore these complex transitions.

The focus in previous research has been on aspects such as the patient’s perspective on the choice of primary care provider [6], the comparison of models across county councils [3] and the impact of financial management control systems on PHCCs [8]. This study seeks to explore how PHCC managers perceived the transition and what impact it had on their work.

In this project, the PHCC manager is regarded as an individual in a group or organisation who has the ability to influence phenomena in the organisation in such a way that shared work can be carried out to achieve healthcare goals. At the same time, he or she is also an individual governed by regulations [10,11]. The potential negative impact of reorganisation and downsizing on the occupational health and safety of employees has been demonstrated previously [12-14]. By managing the inevitable conflicts that arise [15], managers definitely have a key role to play in leading the transition of their unit [16].

### Context

When freedom of choice regarding primary healthcare was introduced in October 2009, the number of PHCCs contracted in the region rose immediately from 143 to 205, of which 200 were still in business one year later [7]. The share of privately owned PHCCs in the region increased from 18% to 42%. These changes led to competition between providers, as the size of the registered population was the principal revenue factor. Many of the publicly owned PHCCs were forced to downsize and transform their organisations. These effects were particularly noticeable in the metropolitan region of Gothenburg, which had the highest number of newly established healthcare centres.

### Aim

The aim of this study was to explore how managers at publicly owned primary healthcare centres perceived the transition of the primary healthcare system and the impact it has had on their work.

## Methods

### Study design and data collection

This is a qualitative study based on interview data using content analysis inspired by Silverman [17]. The COREQ 32-item checklist [18] for qualitative studies was used to assure the quality standards for this study.

The research team included one junior researcher (AM) and three senior researchers (KN, CF, JT), of whom one has published several articles on leadership research. All members of the research team were involved in planning the study and have a professional healthcare background. Two team members work as general practitioners (GPs) in the publicly owned primary healthcare organisation. Initially, the research team wanted to interview managers from both public and private centres. However, private centres refused to participate due to a heavy workload. The participants were selected from 28 publicly owned PHCCs in the metropolitan region of Gothenburg. Two managers at two PHCCs were excluded from the study design as the manager of one PHCC was part of the research team and the manager of the other was working in a PHCC specialized in refugee medicine that was not affected by the system change. The remaining 26 managers were informed at manager meetings that their participation was voluntary. They received a written research plan outlining the purpose of the study and they were told that the results would be presented in a way that would guarantee confidentiality. There were no pre-existing relationships between the interviewers and the participants. Managers were contacted by the interviewers by telephone and were asked if they wished to participate. If they were interested, an appointment was made for a one-hour interview. Two managers exercised their right to withdraw due to a heavy workload. One interview was conducted jointly by the junior researcher (AM) and the senior researcher (KN). The interviews were then conducted individually by these two researchers, 11 and 12 interviews respectively. The interviews were carried out in face-to-face meetings at the managers’ offices and in a setting in which they would not be disturbed. A semi-structured interview guide was developed by the research team and was used in the interviews, which lasted 23–56 minutes. In summary, the interview guide focused on the following research questions:

•How did healthcare managers handle the new situation involving competition among healthcare centres?

•How were daily routines managed after the transition?

•Which opportunities and obstacles arose from the transition in terms of further development of the healthcare centres?

The 24 participants were 43–66 years of age; 20 were female and four were male. Fourteen participants had a nursing background, seven were GPs, two were physiotherapists and one had a background in social work. Many participants had attended various leadership programmes, most of which carried more than 30 higher education credits. The majority had attended programmes that included change management skills. Each PHCC had a patient population of 8,000-20,000. Many of the PHCCs lost a certain percentage of their patients following the transition.

All interviews were audio-recorded and transcribed verbatim by a professional secretary. The quality of the transcripts was checked by the interviewers with regard to consistency with the audio recordings.

### Analysis

The interviews were analysed using a qualitative approach with content analysis inspired by Silverman [17]. Both the manifest content and the latent content were analysed to explore and describe which changes were experienced and how the transition of the primary healthcare system was perceived and managed.

The transcripts were read through several times by AM, KN and CF to gain an overview of the content and to obtain a sense of the whole. Using the NVivo™ 9 program, the research group coded the interviews separately into units of meaning and merged their codes in several rounds of discussions. The manifest content was identified and described, followed by identification and interpretation of the latent content. Categories and subcategories were created, reflecting both the manifest and the latent content, and were provided with quotations. The categories were refined through repeated revision of transcripts and codes in order to ensure consistency with the whole and to explore the various perceptions. Finally, two core themes were identified by the research group. Each interview quotation was given a number (1–24).

### Ethical considerations

According to Swedish law governing ethical review of research involving humans [19], this study did not require ethical approval. Before the interviews, the participants were informed that their participation was voluntary and that they had the right to withdraw at any time without being required to give a reason. Written, informed consent was obtained from all the participants.

## Results

The participants’ descriptions of their experiences regarding the transition of the primary care system and how their work was affected will be described in three categories with additional sub-categories. The first two categories show a clear differentiation into positive and negative perceptions, as described in the subcategories (Tables 1 and 2), whilst the third category does not show this dichotomisation (Table 3). Finally, two core themes are presented.

### Financial incentives as the major driving force

**Table 1:** Financial incentives as the major driving force

**Perception**	**Sub-categories**
Positive experiences	- Move towards effectiveness in processes, time management and costs
	- Assignment, tasks and organisational structure became clearer
	- Employees became more result-oriented
	- Easier to follow up and compare financial results
Negative experiences	- Difficult to foresee the future financial position
	- Difficult to achieve a financial balance
	- Increased administrative workload
	- Strict rules lead to less freedom to develop your own ideas
	- Stressful if rules are unclear
	- Reducing the number of staff is stressful
	- Own research activities neglected
Positive and negative experiences	- New competitors
	- Extensive changes took place promptly

All the participants stated that the new economic incentives are the predominant driving force in all the changes. With the introduction of a new system involving freedom of choice of primary healthcare provider, a ‘book of rules’ was published for all contracted healthcare centres, defining in detail the requirements and rules for payment. The traditional budgetary system was substituted by a system under which healthcare providers received payment based primarily on the number of registered patients and their estimated illness burden. Managers perceived this paradigm shift as positive and they strove to achieve greater efficiency in processes, time management and costs.

*Yes, it’s all about slimming down; streamlining what we have and ensuring that everyone who works here knows the exact nature of their duties and performs them as well as possible. If everybody does what they should be doing, we will achieve great results* (15).

They felt that the healthcare centres’ mission, tasks and organisational structure were better defined. There is a clear link between the assignment and payment. As the mission was more obvious, the manager also needed to demonstrate clearer leadership.

*The greatest effect is that you need to be extremely focused on the financial side. This is the biggest change although I also need to demonstrate more clearly to employees what we are supposed to do and not do. You focus more on making sure you perform the tasks you are paid for – that’s the way we survive* (10).

Furthermore, some managers had the positive experience that employees became more aware of result orientation and the quality parameters. As the transparency of the financial reports makes them detailed and clear it was easier to follow up and compare financial results.

*You have full transparency on the financial side. You have control of the figures; you see where every penny is going./…/Then you know exactly what action you need to take to get your business working* (15).

At the same time, a number of negative effects were experienced by managers, who needed to handle considerably more financial matters than previously. Some managers described the difficulty of acquiring an overview and predicting the future financial position because of shortcomings in their financial training.

*I can’t make an arm’s length assessment of how we would be affected if we had 100 patients more on our list compared to if we lost 100 patients* (14).

These managers also found it difficult to achieve a financial balance and they attributed this to fluctuations in the number of listed patients and a lack of cost control.

*We are separate financial entities, which is new and big for us. It’s kind of scary. What if we don’t get it to balance?* (5).

The increase in the administrative workload can be attributed to the fact that more of the administrative work had been decentralised and transferred to the PHCCs. The managers therefore felt they had lost valuable time dealing with petty, practical administrative work, which they felt was not what a manager should be doing. Instead, some managers preferred to devote more attention to development.

*All these bureaucratic tasks exhaust me. It really eats up a lot of my time. I think all the managers feel the same way* (5).

Managers expressed disappointment as they expected to have more freedom to develop their PHCCs based on their own ideas and to make more of their own decisions following the transition. However, their experience was the opposite and they questioned the role of the management level above them.

*You thought you would have more freedom but it became narrower instead.* (13)*.*

Managers generally experienced the uncertainty in the rules as stressful, such as when it was unclear whether registration at a child health centre (for regular check-ups for children up to the age of five) also meant registration at the associated PHCC.

*The child health centres have also been affected a great deal. It’s been terrible…there was a lot of trouble regarding the child health centres* (12).

Managers found it extremely difficult and stressful to deal with the situation when they were forced to reduce the number of staff for financial reasons while at the same time the staff workload became almost unbearable.

*What I can say in the current situation is that the staffing level is so minimal that we can’t cut back any more. Our financial managers keep saying: ‘You have to cut back on staff. Even though we know they are working themselves into the ground.’ It’s impossible* (19).

Although there was the possibility of applying for research funds, managers stated that research was not a priority.

*We don’t have very much time for research unfortunately.* (1).

Depending on the local situation at the PHCC, managers perceived the new competitors that had become established in their district as completely different. Those who had not made an active choice of healthcare centre were assigned to the PHCC that was geographically closest, which in part led to sudden and enormous changes in the number of registered patients. Managers in deprived areas, where the number of PHCCs and access rates were previously low, welcomed new competitors.

*The task assigned to us was too large. It was impossible to have a district of 23,000 [inhabitants]/…/We couldn’t manage. So it was great that a competitor came into this very deprived area/…/It was good for us* (26)*.*

However, managers in central districts, which was where the majority of new, privately owned PHCCs had opened, considered it stressful and difficult to handle a new situation with noticeable competition.

*I was concerned because we were exposed to incredible competition here in the [city] centre./…/I was worried about how we would balance our finances and that many [employees] would need to be fired* (11)*.*

Generally, it was felt that extensive changes could take place very quickly due to competition and financial incentives but the consequences were judged differently. Some managers regarded it as a positive challenge.

*Yes, we’ve learned … we understand what is most important. You have to be on the lookout so that you can change in advance./…/This is not an easy nut to crack.* (17).

In other cases, when the result was staff reductions or a higher turnover of staff, managers spoke about their distress at the loss of old structures. This situation was considered turbulent. Some of the staff chose to leave the centre and move to a privately owned centre. The staff who chose to stay then had to cope with the resulting despondency.

### Shift of power from the healthcare provider to the ‘customer’

**Table 2:** Shift of power from the healthcare provider to the ‘customer’

**Perception**	**Sub-categories**
Positive experiences	- Access to PHCCs became easier and faster through drop-in receptions
	- More welcoming, friendly and communicative attitude to patients
Negative experiences	- Unreasonable, demanding patients
	- Reduction in the number of planned visits for chronically ill patients in favour of time devoted to minor complaints
	- Loss of home visits
Positive and negative experiences	- The shift of power is leading to prioritisation conflicts

One aim of the transition was to shift power from the healthcare providers to the patients. Patients acquired a great deal of power by having the opportunity to choose their favourite PHCC, which led to rises and falls in the PHCCs’ revenue flows.

Managers described a clear shift of power in favour of the patients and took a positive view of the change as access to a PHCC became easier through the introduction of drop-in receptions.

*We never had a drop-in reception before and now we have it every day between 9 and 3. We have a much better access rate than before* (10)*.*

The attitude towards the patients became more welcoming, friendly and communicative. The patient was no longer seen as simply someone who needed to see a doctor but as a customer of the PHCC. At the same time, managers described the negative effects of this shift of power. Patients could become unreasonably demanding and ask for a GP even if their complaint could be handled by a nurse or another medical professional. The same applied to the demand to be referred to a specialist even if the complaint could be handled at the PHCC. This implied an overuse of the medical services and inefficient utilisation of competences and resources.

*I find that patients have become so demanding: I’ve read this…, I found this on the internet…, I want this examination…, I want to see the doctor immediately…* (6).

Managers explained that what was previously a major part of their work, i.e. planned visits for patients with chronic diseases, who were often multi-morbid individuals who sometimes required home visits, had been reduced in favour of drop-in receptions used mainly by patients who were generally healthier and who attended with minor complaints.

*We need to devote so many resources to these drop-in receptions. I feel we see a lot of patients who don’t really need to come to us* (11).

The managers stated that the shift in power is leading to conflicts in prioritisation. The shift in power to the patient does not automatically imply better quality of care. Even if easier access and a more welcoming attitude can be regarded as an improvement in quality for the patient, the transfer of resources to drop-in receptions could reduce the time available for patients with more complicated complaints and who require longer consultations. Easier access is perceived as a driving force against patient empowerment and the capacity for self-care of simple conditions.

*A chronically ill [patient] gets to meet a doctor for two or three minutes. This is really the wrong forum. Many patients are dissatisfied with the visit and need to return./…/The patient is given too much power to control something that actually leads to worse care* (15).

### Shortcomings in management skills

The managers interviewed were asked which strategies they used to manage the extensive changes that were required. All the managers stated that they were offered external support through a company contracted by the board of directors. The majority of the managers collaborated with the external consultants and stated that it was mainly the consultants who led the change management process. Some managers also expressed a need for more support and consultation due to shortcomings in their change management skills.

*I thought many times that it was a really tough year and I wished I had been given a bit more training* (11).

When asked to name concepts or strategies to manage change, only a few managers could name such strategies. The majority of the participants were not able to describe their concepts in more detail than was the case during the repeated discussions that took place with team members regarding tasks and financial issues as part of managing the transition. Some managers felt that they lacked training in the administrative and financial skills that were required following the transition and they felt it was difficult to remain updated on changes in regulations.

*We try and take small steps forward but as I see it we have no structured system to develop the organisation…* (4).

The change management process overall was regarded as being very stressful as changes needed to be made swiftly, including reducing and transferring staff.

*I’m really concerned about the activities here, really worried. I feel mentally worse.* (12).

### Core themes

An overall perspective on the interview material and the results from the analysis resulted in two core themes.

#### The transition is perceived as a rapid change, enforced mainly through financial incentives

Noticeably, all managers felt that the transition of the system made financial issues their main task with effects that were considered to be both positive and negative. On the one hand, financial incentives were perceived as positive – they were a driving force and a tool for change. On the other hand, financial issues became a stress factor for managers due to uncertainty, competition with other PHCCs and negative feelings associated with reducing the number of staff. The differences in perception were influenced by preconditions, such as the number of local competitors and the managers’ change management and financial skills. The general consensus was that the changes took place rapidly.

#### Prioritisation conflicts arise between patient groups with different needs, demands and levels of empowerment

The shift in power towards the patient, which was considered both positive and negative, gave rise to a new conflict: difficulty prioritising correctly among patient groups with differing needs, demands and levels of empowerment. The managers prioritised drop-in receptions and noticed a positive change through improved access and service for the population. At the same time, managers expressed frustration due to the increase in the number of patients with unreasonable demands. Furthermore, they were concerned about the negative shift in PHCC usage towards mostly healthier individuals. They were finding it difficult to provide adequate follow-up for less empowered patients with more extensive needs, mostly multi-morbid patients.

## Discussion

The transition of the primary care system in western Sweden had an extensive impact on the publicly owned PHCCs. This qualitative study shows that the new financial incentive system in particular led to rapid and powerful changes and these were perceived and handled differently by PHCC managers. Their perception was influenced by differing preconditions related to the competitive situation locally as well as variations in management skills. The major effects of the transition described by the managers have also been demonstrated in a number of other studies [3,20,21], i.e. the shift in power towards the patient as well as the increase in the number of PHCCs with a resulting increase in competition. The improved access and level of service for the patients were generally perceived as positive by the managers but they also expressed concern and frustration about associated issues, such as overly demanding patients and prioritisation conflicts between patient groups with different needs, demands and levels of empowerment. This shows clearly the complexity of the system change and the importance of improved follow-up and means of control in order to monitor and govern system changes and ensure development towards a more effective and sustainable primary healthcare system. If power shifts too much in one direction, it may result in an imbalance, with unwanted and costly effects that could unintentionally lead to a more ineffective primary care system.

A recent quantitative study [22] confirmed the PHCC managers’ perceptions identified in this qualitative study. This study examined the transition in three other Swedish counties and showed that the number of visits increased within all patient groups and that the increase was higher for patients with simple complaints compared to patients with more complex medical problems. Bearing in mind that managers stated that the latter could also receive short consultations at drop-in clinics that were not suited to their requirements, it is questionable whether the increase in the number of visits has resulted in greater benefit for these patients. In contrast to these trends, the Swedish National Board of Health and Welfare recommends the development of local health and social care programmes to meet the demands of older individuals [23]. Other factors, such as interpersonal continuity, need to be taken into account to study the value of the transition for this vulnerable group, which accounts for the largest proportion of costs in primary healthcare [24].

Another finding in the quantitative study [22] mentioned above is that patients with higher socioeconomic status reported a level of satisfaction with access to primary care that was higher than the average for the population. At the same time, another study found that the number of new establishments, and thus more competition, was highest in centrally located metropolitan districts and in high-income areas [25]. Managers in these districts experienced most stress due to the transition because of their challenging financial situation, revealing that the satisfaction gained in this group came at a cost.

In the transition, the request for rapid change came from political quarters and was accomplished successfully through financial incentives although the effects need to be monitored carefully over time to ensure the organisational changes are sustainable. According to Gersick’s revolutionary change theory, this transition is equivalent to a brief period of revolutionary upheaval after a long period with a stable infrastructure and with only incremental changes [26]. The perceived difficulties and conflicts that arise from making the correct prioritisation between financial constraints and patient demands are an indication of what Gersick describes as a change in the ‘Deep Structure’. This conflict of values may affect the degree of innovation needed to meet changing care demands, such as the rise in the number of patients with multiple chronic diseases or mental health problems. Previous research has shown that these innovations often arise through internally driven initiatives and not through external financial incentives [27,28]. Future investigations must ensure that necessary organisational innovations are not suppressed by changes in the Deep Structure resulting from rules and regulations. An indication of this could be the fact that managers expressed a lack of interest in participating in medical research projects despite the availability of research funding.

### Shortcomings in change management skills

**Table 3:** Shortcomings in change management skills

**Perception**	**Sub-categories**
Negative experiences	- Lack of concepts and strategies. Although managers received support some felt a need for more support due to shortcomings in their change management skills.
	- Managers lack training in the administrative and financial skills required following the transition.
	- Managers have difficulty remaining updated on changes in regulations.
	- Managers feel mental pressure due to changes that needed to be carried out swiftly, including reducing or transferring staff

The managers’ leadership problems described in this transition are caused primarily by shortcomings in change management skills and uncertainties in the new system, including the number of future competitors, thus giving the impression that managers have to a certain extent lost control of their change processes. One possible approach when addressing such issues in future transitions could be the establishment of communication platforms and frequent meetings between the governance administration and PHCC managers. Feedback loops have the potential to detect unintended developments and distress at an early stage. The pace and direction of transitions could be controlled by making adjustments in the regulations, e.g. restricting extreme oversupply in one district in favour of a more equal establishment of new PHCCs in all districts. However, the dynamics of feedback loops in public sector organisations are complex and could even produce instability [29]. The support that managers received during the transition focused mainly on the business process although the fact that managers asked for more support and expressed distress were an indication of a need for more consulting in leadership issues.

It must be pointed out that this study is limited to the perceptions of the managers of publicly owned PHCCs in the metropolitan area of Gothenburg, where the managers had different professional backgrounds and the majority were female nurses. Even if this is representative of how publicly owned PHCCs are commonly managed in Sweden, the metropolitan area of Gothenburg is not representative of how reforms in Swedish primary care have influenced publicly owned PHCCs in general. Previous studies with data from primary care have suggested that nurse managers are more loyal to organisational objectives and organisational control compared to managers who are doctors [30]. However, in this study the research team could not identify tendencies or differences in perception based on the professional background of the managers. Further studies that include different medical professions are necessary to gain a more complete perception, including the effects on continuity and quality of care. The study is also limited by the exclusion of privately owned PHCCs and PHCCs located in more remote areas, making it impossible to draw conclusions for the whole of western Sweden.

However, a strength of the study is that we have been able to explore in depth the perceptions of this selected group with similar preconditions. A wide range of preconditions at different centres may have blurred the results. The major challenge that remains when making future adjustments in the recently introduced system is to find the correct balance of power between healthcare providers and patients in order achieve a sustainable system. Other future transitions, including the introduction of freedom of choice of rehabilitation provider or secondary care provider, could profit from infrastructures such as frequent feedback loops and instant access to leadership consulting that help to monitor and adjust the pace and direction, even during the transition process, and thus reduce unintended effects.

## Conclusions

This qualitative study shows the complexity of the system change and describes the different effects and perceptions of the transition from a PHCC manager’s perspective. The transition was perceived as rapid, enforced mainly through financial incentives and leading to prioritisation conflicts between patient groups with different needs, demands and levels of empowerment. It suggests a need for improved follow-up and control in order to monitor and govern system changes and ensure development towards a more effective and sustainable primary healthcare system.

## Competing interests

The authors declare that they have no competing interests.

## Authors’ contributions

AM: study design, data collection, analysis, preparation of the manuscript. KN: study design, data collection, analysis, consistency in the manuscript. CF: study design, analysis, consistency in the manuscript. JT: study design, consistency in the manuscript. All authors read and approved the final manuscript.

## Pre-publication history

The pre-publication history for this paper can be accessed here:

http://www.biomedcentral.com/1472-6963/13/382/prepub
